# PrimerView: high-throughput primer design and visualization

**DOI:** 10.1186/s13029-015-0038-2

**Published:** 2015-06-04

**Authors:** Damien M. O’Halloran

**Affiliations:** Department of Biological Sciences, The George Washington University, Science & Engineering Hall 5685, 800 22nd St N.W., Washington, DC 20052 USA; Institute for Neuroscience, The George Washington University, 636 Ross Hall, 2300 I St. N.W., Washington, DC 20052 USA

**Keywords:** PCR, Primer design, Genotyping, Perl, NGS, Sequencing

## Abstract

**Background:**

High-throughput primer design is routinely performed in a wide number of molecular applications including genotyping specimens using traditional PCR techniques as well as assembly PCR, nested PCR, and primer walking experiments. Batch primer design is also required in validation experiments from RNA-seq transcriptome sequencing projects, as well as in generating probes for microarray experiments. The growing popularity of next generation sequencing and microarray technology has created a greater need for more primer design tools to validate large numbers of candidate genes and markers.

**Results:**

To meet these demands I here present a tool called PrimerView that designs forward and reverse primers from multi-sequence datasets, and generates graphical outputs that map the position and distribution of primers to the target sequence. This module operates from the command-line and can collect user-defined input for the design phase of each primer.

**Conclusions:**

PrimerView is a straightforward to use module that implements a primer design algorithm to return forward and reverse primers from any number of FASTA formatted sequences to generate text based output of the features for each primer, and also graphical outputs that map the designed primers to the target sequence. PrimerView is freely available without restrictions.

## Background

With the advent of next generation sequencing (NGS) technologies, there has been an explosion in the volume of genomic data available to researchers. NGS provides a platform to rapidly sequence genomes, and offers new ways to unlock the genomes of species that are difficult to maintain. Creative federal incentives in the US (National Human Genome Research Institute - http://www.genome.gov/10000368) have contributed to an unprecedented drop in the costs involved in sequencing a genome from ~ $100,000 in 2002 to ~ $5000 in 2013 [1], which in effect has converted a field that was previously dominated by consortiums into an open playing field where small individual labs can participate. However, to prevent individual researchers from becoming caught in the maelstrom of this new genomic era, it is imperative to develop open source and user-friendly tools to help investigators study this volume of data. Designing primers to validate candidate genes from RNA-seq projects as well as developing diagnostic tools for the genomes of recently sequenced species, are examples of routine tasks faced by researchers in tackling NGS related data. Primer design, and in particular primer design *en masse*, also becomes essential for researchers working with multi-gene families, or metagenomic samples [2], as well as many other PCR based applications including primer walking, assembly PCR, digital PCR, ligation PCR, nested PCR, and quantitative PCR. Therefore, as the volume of genomic data continues to increase, so does the scale of experiments related to its analysis, and this is particularly true for primer design.

Here I describe a Perl module called *PrimerView* that is straightforward to implement or plug into larger pipelines, and enables the user to automate the process of primer design for DNA datasets of any size. Often, a visual readout of primer position on the target sequence is the fastest and most helpful way to validate the distribution and position of primers, and to this end PrimerView includes graphical outputs for each primer mapped to its target sequence. Each primer/target sequence pair is aligned and converted into a JPEG formatted file for easy visualization (other formats are also available). A PNG format file is also generated by PrimerView to depict the distribution of all designed primers across each input sequence. PrimerView uses the popular Bioperl [3] modules to align primers to the target sequence using the alignment software MUSCLE [4], and also to convert the alignment from CLUSTAL [5] format into graphical files. PrimerView may be particularly helpful for researchers working with large datasets where primers must be efficiently designed for many genes, as well as for various PCR applications including primer walking and assembly PCR reactions, where a graphical output can quickly help users determine primer coverage and distribution.

## Implementation

PrimerView is written using Perl and has been tested successfully on both Windows command prompt as well as UNIX. PrimerView uses the Bioperl [3] dependencies Bio::Align::Graphics, Bio::Graphics, and Bio::SeqFeature::Generic to generate graphical output, which are all freely available from CPAN (http://www.cpan.org/). The alignment software MUSCLE [4] is used for a single iteration to map each designed primer to the inputted sequence, and this alignment is then converted into JPEG and PNG images depicting the position and distribution of all primers across each inputted sequence. PrimerView is a package with a constructor subroutine called “new” that allows the user to run the module by instantiating a PRIMERVIEW object. Separate subroutines for primer design, alignment, and conversion to graphical output, are called from a script called ‘primerview_driver.pl’. The input for PrimerView is any number of sequences in FASTA format [6]. A sample sequence file called ‘test_seqs.fasta’ is included in the download. The main primer design subroutine of PrimerView requires various parameters, which can be collected from the command-line. Default settings for each parameter will be invoked in the absence of command-line arguments, with the exception of the input filename which must be provided. These options (a through to k) are as follows: [−a filename *e.g.* test_seqs.fasta] [−b 5′ search area, integer] [−c 3′ search area, integer] [−d primer length max, integer] [−e primer length min, integer] [−f GC clamp Y or N] [−g upper GC%, integer] [−h lower GC%, integer] [−i upper Tm, integer] [−j lower Tm, integer] [−k specificity to the entire input file (Y) or just the specific sequence (N), Y or N]. The ‘-b’ and ‘-c’ flags refer to the five prime or three prime search areas across which PrimerView will scan for appropriate primers within each sequence; if the user wants to scan the entire length of the sequence, these flags can be set to the total sequence length in nucleotides. Features of the basic algorithm for PrimerView have been described previously and use nearest neighbor thermodynamic calculations to determine primer *T*_*m*_ values [7–9]. Example settings to execute PrimerView are: “ > perl primerview_driver.pl -a test_seqs.fasta”.

## Results and discussion

**Fig. 1:**
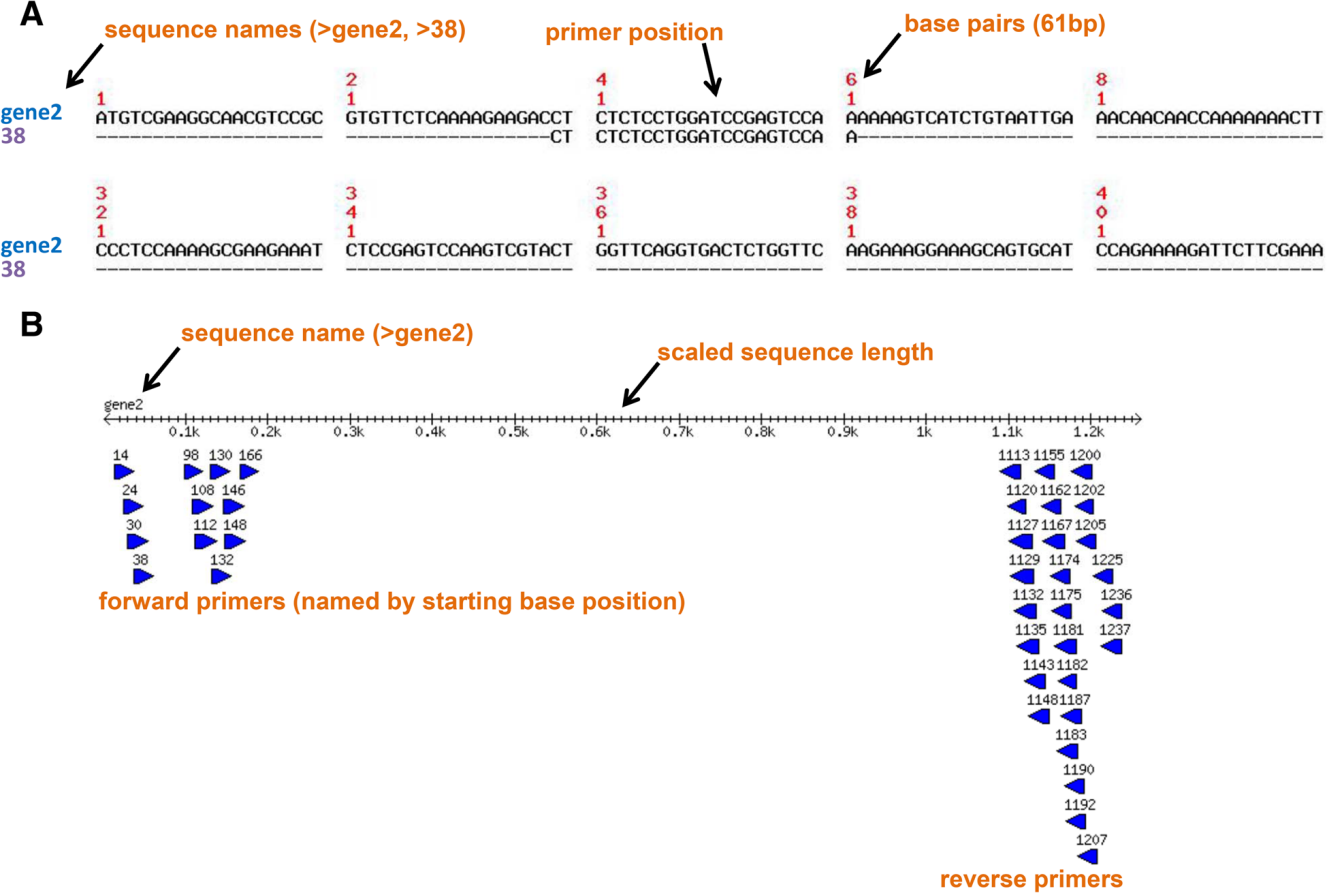
Screen-shot of the graphical outputs generated by PrimerView. **a** PrimerView designs primers for datasets of any size and generates a JPEG format file of the alignment for each primer illustrating its position on the target sequence. The user inputted sequence name (derived from the FASTA description line) is shown as well as the base pair positions and location of the designed primer. **b** A PNG file is also generated by PrimerView that maps the distribution of all designed primers onto the scaled target sequence. The target sequence is represented by a number line that equates to nucleotide position. The gene name is indicated above the number line and the name of each primer is represented by its starting position in nucleotides

PrimerView is an easily implemented Perl package that automates the design of forward and reverse primers from datasets of any size, while generating graphical output in JPEG and PNG formats for each designed primer mapped to the target sequence. For the JPEG generated file, other output formats are available for the graphical output by simply changing the output file extension in the ‘graphic’ subroutine within ‘PRIMERVIEW.pm’ to ‘png’ or ‘gif’ (see CPAN page for Bio::Align::Graphics for more details - http://search.cpan.org/~cjfields/BioPerl/Bio/Align/Graphics.pm). The function performed by PrimerView provides automation of a routine task, with user-defined features to provide more custom usage, especially in larger pipelines. By generating plots that map each designed primer against the target sequence provides easy and fast validation controls for researchers to examine the distribution and position of each primer. Figure 1a shows a representative image from the conversion of a MUSCLE program derived alignment in CLUSTAL format into a JPEG file to generate a primer map for a designed primer to the target sequence. The sequence name is provided in the user input file as the FASTA format header line, and a primer name that refers to its start position in base pairs on the target sequence is also highlighted, as well as the position of the primer. Figure 1b depicts an example output from PrimerView of the PNG file generated, illustrating the distribution of each primer mapped to the scaled input sequence. The primers are denoted as arrowed glyphs pointing in the direction of synthesis with each primer named by its starting position.

**Fig. 2:**
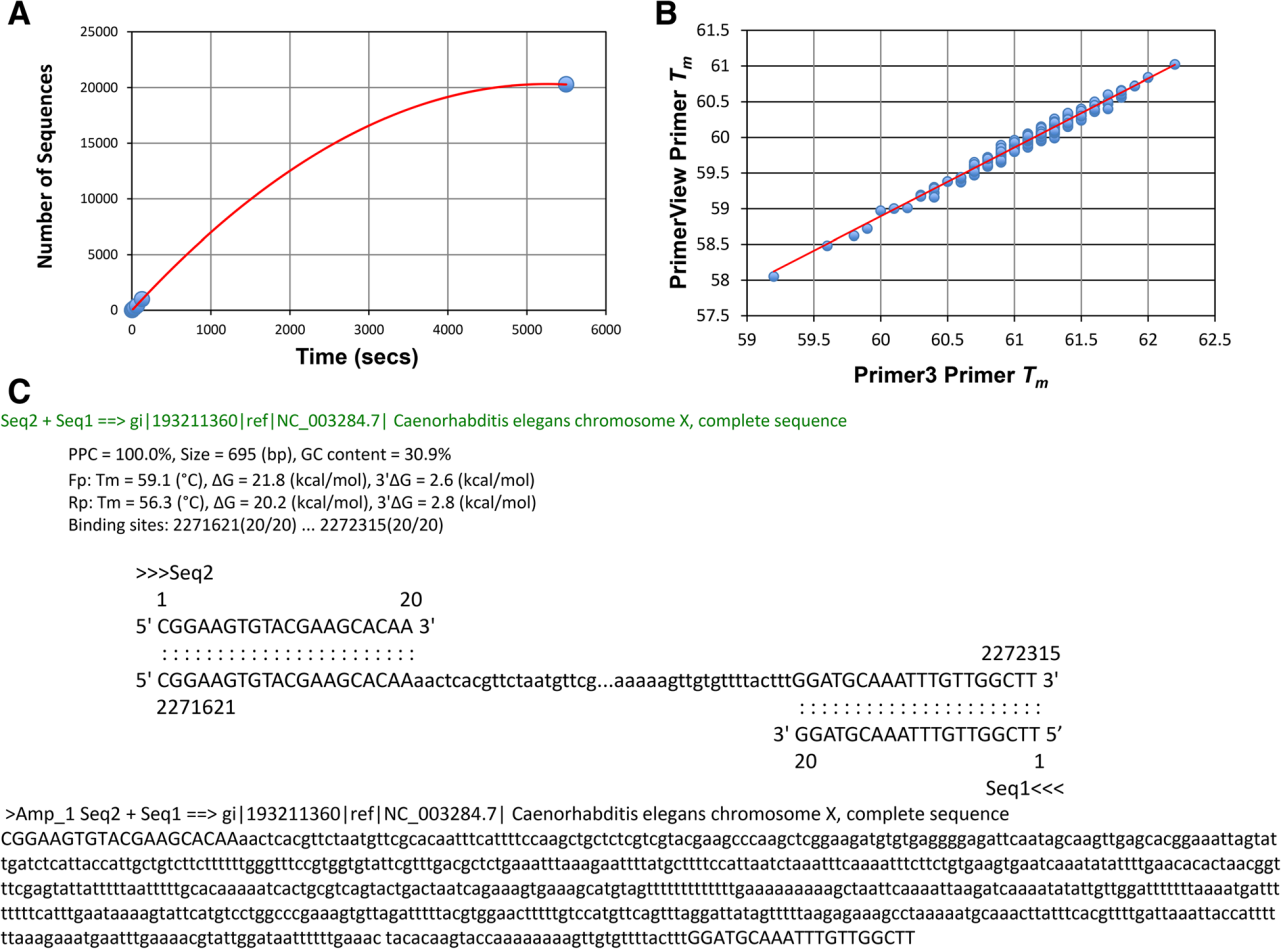
Testing and validation of PrimerView. **a** Performance testing of PrimerView. Files containing different numbers of sequences (10, 20, 50, 100, 200, 400, 1000, 20,284 – obtained from WormBase ftp site: ftp://ftp.wormbase.org/pub/wormbase/) were provided as input to PrimerView and ran using default settings to generate PNG file primer distribution outputs. Fitting the relationship between sequence number and run-time with a quadratic equation yields an R^2^ value of 1.00. **b** Comparison of primer *T*_*m*_ values between PrimerView with Primer3 [10]. To perform these tests only the *calcTm* subroutine within PrimerView was called for 400 primers designed using Primer3 [10]. A robust correlation (R^2^ value of 0.97) was observed between each method, both of which employ nearest-neighbour parameter sets. **c** Specificity and orientation of primers returned using PrimerView was tested using the MFE-Primer 2.0 *in silico* PCR tool (http://biocompute.bmi.ac.cn/CZlab/MFEprimer-2.0/) [11, 12]. Sample output from one test showing the sequence file (NC_003284.7) in FASTA format, and displaying all sequence matches in the database that lie between and include the primer pair (Seq2 and Seq1). The FASTA header details the region in the database as well as the primers; the body of the FASTA sequence is capitalized in areas where the primer sequence matches the database sequence and in lower-case elsewhere

During testing and validation of PrimerView, files containing varying numbers of sequences (10, 20, 50, 100, 200, 400, 1000, and 20,284) were provided as input to PrimerView, and ran using default settings to generate PNG file primer distribution output. The output from these performance tests is shown in Fig. 2a by plotting the program run-time for files containing increasing numbers of sequences (y-axis) against execution time in seconds (x-axis). A linear fit (m*x + b) reveals an R^2^ value of 0.9994, however, fitting the relationship with a quadratic equation (a*x^2 + b*x + c) yields an R^2^ value of 1.0. Testing also included a comparison between the primer *T*_*m*_ values returned from PrimerView to that of Primer3 [10] (Fig. 2b). To perform this test only the *calcTm* subroutine within PrimerView was called for 400 primers designed using Primer3 [10]. A robust correlation (R^2^ value of 0.97) was observed between each method, both of which employ nearest-neighbour parameter sets. Finally, validations of numerous primer pairs returned using PrimerView were tested using the MFE-Primer 2.0 *in silico* PCR tool (http://biocompute.bmi.ac.cn/CZlab/MFEprimer-2.0/) [11, 12]. An example output from one primer pair validation test is shown in Fig. 2c, and in all test cases each primer returned from PrimerView exhibited correct orientation and specificity.

## Conclusions

By handling both single sequence and multi-sequence input, PrimerView facilitates automated primer design for specific targets as well as large gene datasets. Although many other primer design tools exist such as Primer3, BatchPrimer3, and PerlPrimer [10, 13, 14], the utility of PrimerView are the graphical outputs that can quickly and easily depict the distribution of all primers across a target sequence from multi-sequence input. Generating graphical outputs that map each designed primer to the target sequence is an efficient means of quickly validating the spread of primers across a target.

## Availability and requirements

- Project name: PrimerView
- Project home page: https://github.com/dohalloran/PrimerView
- Operating system(s): Platform independent
- Other requirements: Bioperl
- Any restrictions to use by non-academics: None
